# Development of an Online General Biology Open Educational Resource (OER) Laboratory Manual

**DOI:** 10.1128/jmbe.00133-21

**Published:** 2021-07-30

**Authors:** Dmitry Y. Brogun, Azure N. Faucette, Kristin Polizzotto, Farshad Tamari

**Affiliations:** a Department of Biological Sciences, Kingsborough Community College, The City University of New York, Brooklyn, New York, USA

**Keywords:** General Biology, OER, online lab manual

## Abstract

Currently, many academic institutions are using one or more variations of online modalities due to the COVID-19 pandemic, and science educators face a unique challenge with distance-learning laboratories. Many resources to engage students in virtual, interactive laboratory activities exist, but we found that high costs and/or overlooked content left gaps for several topics typically taught in a general, introductory biology course for undergraduate biology majors (e.g., organismal biology). Additionally, resources for an online lab must be identified and curated from multiple sources, requiring intense demands on the instructors’ time. To meet this need and to overcome the financial burden of high-cost lab manuals or software, we developed, piloted, and revised a series of online general biology lab exercises. We have published these exercises as an Open Educational Resource (OER) digital laboratory manual under the Creative Commons License Agreement, and they are accessible online via Manifold, Creative Commons, and the CUNY Academic Works portal.

## INTRODUCTION

The COVID-19 pandemic and the resulting switch to remote learning have complicated two major challenges faced by many undergraduate students and their instructors: (i) the high cost of traditional lab manuals and (ii) the availability of online laboratory exercises. As the spring 2020 semester transitioned from fully in-person to fully online courses, we began searching for open educational laboratory exercises. We searched for high-quality, interactive activities that aligned with the learning outcomes for our general biology courses for biology majors (see the learning outcomes for General Biology I [https://www.kbcc.cuny.edu/academicdepartments/bio/biosyllabus/Bio-13-Syllabus-2021.pdf] and General Biology II [https://www.kbcc.cuny.edu/academicdepartments/bio/documents/Bio_14_syllabus-2021.pdf]) and that could be completed by students working asynchronously.

General biology typically includes the scientific method, microscopy, biochemistry, cell structure and function, enzymes, photosynthesis, cellular respiration, and genetics, and there are some high-quality, virtual Open Educational Resource (OER) labs available for these topics (for example see the Table of Contents for virtual labs in general biology courses at the New York City College of Technology [https://openlab.citytech.cuny.edu/bio-oer/]). However, we found little in the way of fully developed, virtual OER labs for additional topics that often occur in general biology, such as evolution, ecology, and organismal biology, which could be completed independently by students without synchronous instructor guidance. There are excellent interactives available for specific topics in evolution and ecology (for example, from the Howard Hughes Medical Institute’s Biointeractives [https://www.biointeractive.org/]), but these generally require instructor guidance. Open educational resources, including comprehensive, online general biology laboratory manuals exist (e.g., Lumen Learning’s Biology I [https://lumenlearning.com/courses/biology-i-laboratory-manual/] and Biology II [https://lumenlearning.com/courses/biology-ii-laboratory-manual/] Laboratory Manuals), but these are designed for in-person labs rather than a fully online laboratory course. Many high-quality interactive virtual labs have been developed (e.g., Labster, [https://www.labster.com], SimBio [https://simbio.com/]), but these can be expensive, often have hardware requirements beyond what is available to many college students, and are not open resources. Other popular, free content for virtual biology labs is no longer available (for example, see notices of retired virtual labs at the Biology Corner [https://www.biologycorner.com/worksheets/virtual_labs_glencoe.html] and PHSchool [https://www.savvas.com/index.cfm?locator=PS3g2v]). In light of this, we embarked on the development of a comprehensive, fully online, and openly licensed laboratory manual for a second-semester general biology course (see Table 1). This should provide faculty and students with a general biology course that covers evolution, ecology, and organismal biology as a one-stop source for interactive, virtual “lab” activities that can be completed independently and asynchronously by first-year undergraduate students (or advanced high school students).

**TABLE 1:** Lab manual Table of Contents illustrating the topics

Lab topic	Learning objectives
Evolution: Geological Time, Primate and Human Evolution, and Molecular Evolution	Identify major geological and evolutionary events Create a scaled timeline of major evolutionary events and indicate the approximate date of each Calculate the proportion of earth’s history for which various groups of organisms have existed List derived characteristics of primates and humans Distinguish between primitive and advanced characteristics in primate facial and skull bones Analyze evolutionary relationships using molecular (DNA) evidence
Evidence of Evolution	Describe evidence of evolution that is based on microevolution and population genetics Define and use the terminology of population genetics correctly Use the equations of the Hardy-Weinberg equilibrium to calculate allele and genotype frequencies Graph allele frequencies using Microsoft Excel and identify changes in allele frequencies Draw graphs and explain three types of selection
Taxonomy and phylogenetics	Explain how the following evidence is used for phylogenetic reconstruction: the fossil record, DNA, and biogeography. Describe specific examples of phylogenetic reconstruction, such as the relationship of humans to other primates. Build and analyze a phylogenetic tree, identifying patterns of shared ancestry. Differentiate between the allopatric and sympatric modes of speciation.
Bacteria	Identify and define common bacterial shapes and features, including: cocci, bacilli, spirilli, pili, capsule, spore, fimbriae, flagella, plasmid, Gram-positive cell wall, Gram-negative cell wall. Describe various bacterial metabolic processes, including: photosynthesis, chemosynthesis, methanogenesis, nitrogen fixation. Identify and describe at least three vital roles that bacteria play in their ecosystems, such as primary production, decomposition, nitrogen fixation, and disease.
Protista	Define the term “protist” and explain why this is not a monophyletic group. Identify representatives from each supergroup Excavata, “SAR” clade, Archaeplastida, and Unikonta Draw a phylogenetic tree for the eukaryotes and explain why the eukaryote supergroups form a polytomy. Indicate the position of plants, animals, and fungi on the eukaryote tree, and identify the group of protists most closely related to each. Give examples of protist species from each eukaryote supergroup. Give two examples of the significant impact of specific protists on their ecosystems.
Fungi	Describe fungal classification into phyla, and provide a phylogeny of Kingdom Fungi. In a sentence or two, describe the characteristics of the three largest phyla in the Kingdom Fungi (Zygomycota, Ascomycota, Basidiomycota). Using images, explain the life cycle of typical multicellular fungi. Give three examples of how humans benefit from specific uses of fungi
Plants I: Seedless plants	Distinguish members of Kingdom Plantae from their nearest relatives (charophyte algae) Draw a basic phylogeny for Kingdom Plantae Diagram and explain the life cycle of plants (alteration of generations) Differentiate the characteristics of nonvascular plants vs seedless vascular plants Using images, explain the life cycles of moss (a nonvascular plant) and ferns (a seedless vascular plant)
Plants II: Seed plants	Describe the alternation of generations life cycle in plants List characteristics of gymnosperms and angiosperms Identify reproductive structures in gymnosperms and angiosperms Summarize differences between monocots and eudicots Label the reproductive and non-reproductive structures of a flower
Animals I: Invertebrates	Distinguish members of the Kingdom Animalia from their closest living relative (Choanoflagellates and Fungi). Explain the basic body plan of members in the Kingdom Animalia. Identify members of the Phyla Porifera, Cnidaria, Platyhelminthes, Rotifera, Annelida, Mollusca, Nematoda, Arthropoda, Echinodermata. Compare two types of invertebrate life cycles. Compare the structure and function of invertebrates.
Animals II: Vertebrates	List characteristics found in the Subphylum Vertebrata of Kingdom Animalia List characteristics of each of the major tetrapod groups: amphibians, reptiles, birds, mammals; and provide examples of each Identify homologous structures in vertebrates, and explain the functions of each structure Identify representatives from the eight vertebrate clades, Agnatha, Chondrichthyes, Osteichthyes (comprised of Actinopterygii and Sarcopterygii), Amphibia, Reptilia, Aves, and Mammalia Identify and list 11 organ systems in vertebrate animals, their main organs, and provide the major function(s) of each (integumentary, skeletal, muscular, nervous, endocrine, digestive, respiratory, cardiovascular, lymphatic/immune, urinary, & reproductive) Compare the life cycles of amphibians and mammals Identify structures in dissected specimens of representative vertebrates (frog and fetal pig)
Ecology I: Biomes, Population Growth and Predator-Prey Dynamics	Identify the characteristics of Earth’s major terrestrial biomes and describe the impacts humans have had on these biomes. Explain the relationships between climate (temp and precipitation) and terrestrial biome type. Apply the concepts of biotic potential and environmental resistance to human population growth. Explain the difference between exponential and logistic growth and define carrying capacity. Identify major events that have affected human population growth, and explain how they have increased carrying capacity. Explain the dynamics in population size in a real-life predator-prey relationship. Explain the difference between density-dependent and density-independent factors that affect population growth. Interpret real-life predator-prey population data as depicted in a graph.
Ecology II: Community and Ecosystem Dynamics	Explore the concept of an ecological niche and the difference between a fundamental niche and a realized niche using an example of two barnacle species competing for the same resource. Using the same two competing barnacle species, demonstrate how limiting factors (predation and desiccation tolerance) interact to result in competitive exclusion, resource partitioning and realized niches. Investigate the concept of trophic cascades, and explain how a keystone species can indirectly affect the biodiversity and nutrient cycling of an entire ecosystem.

**FIG 1:**
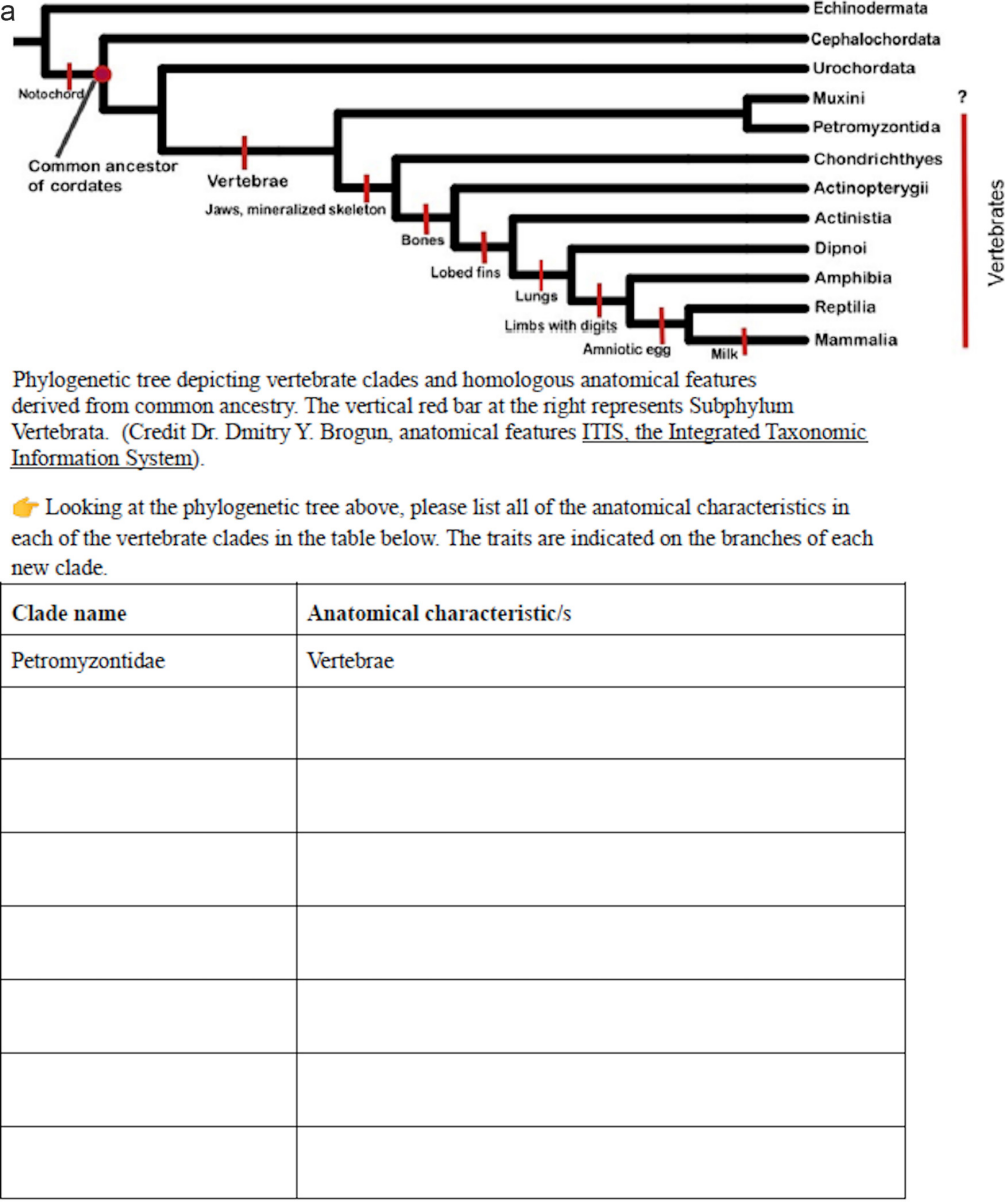

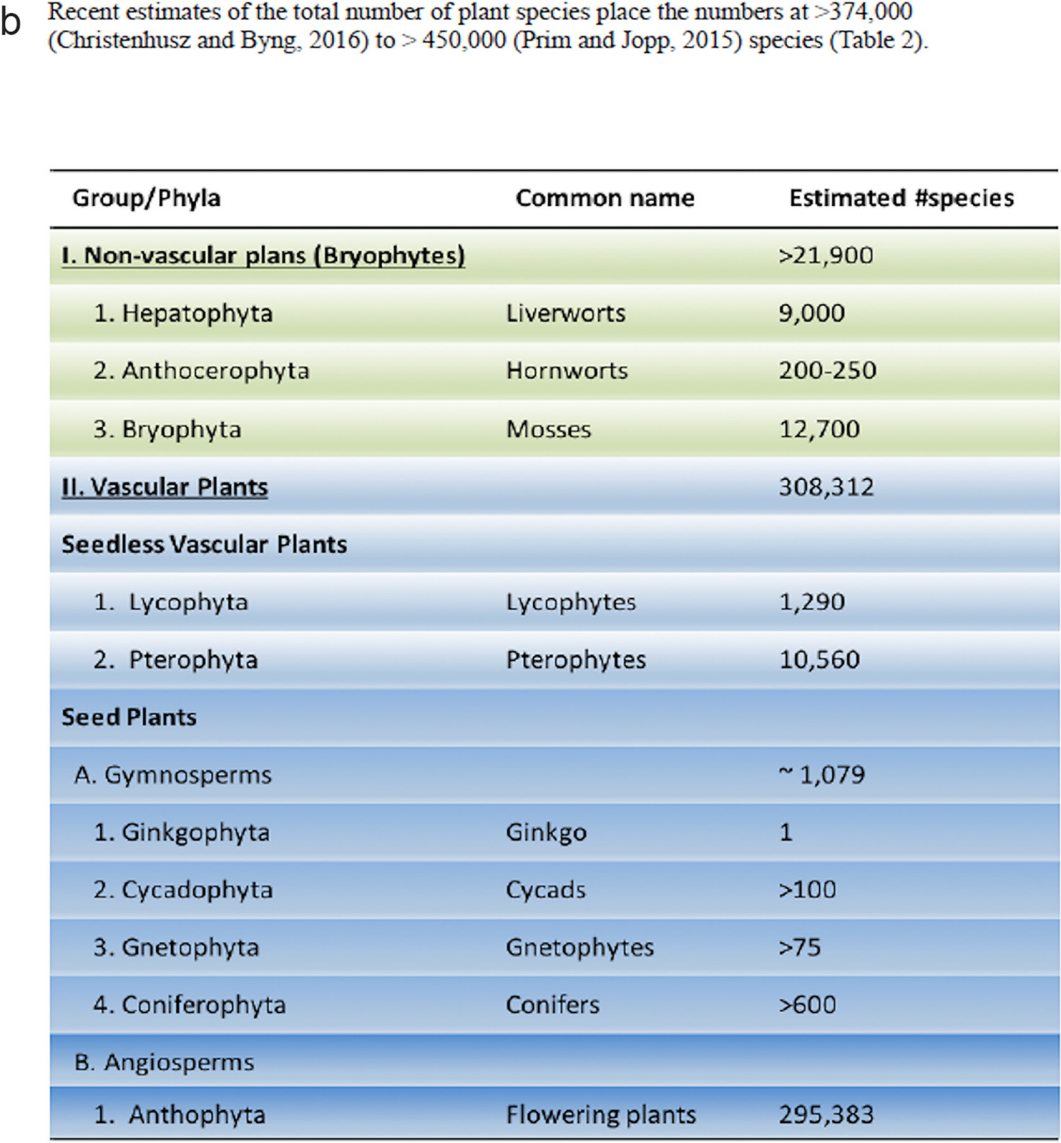

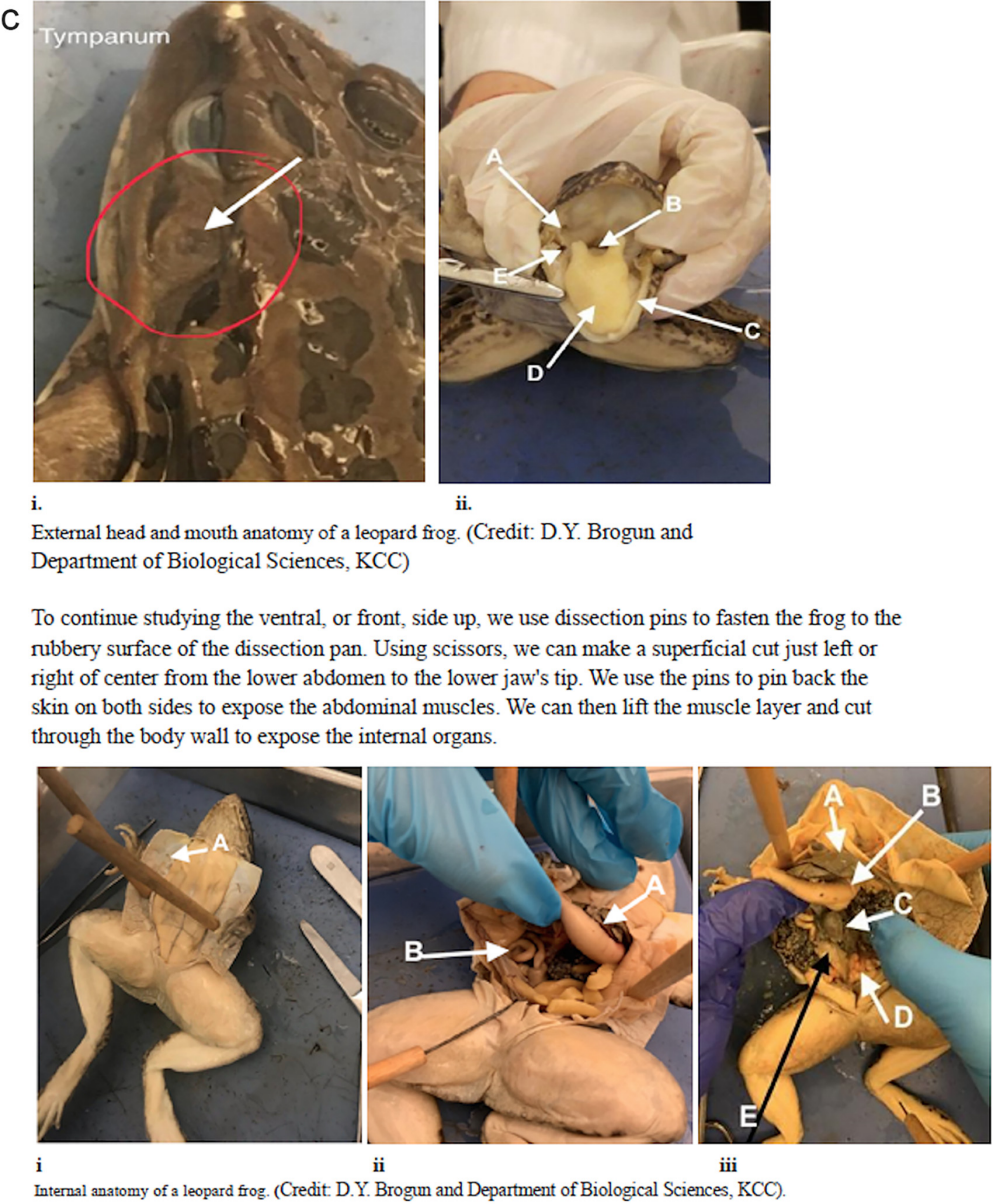

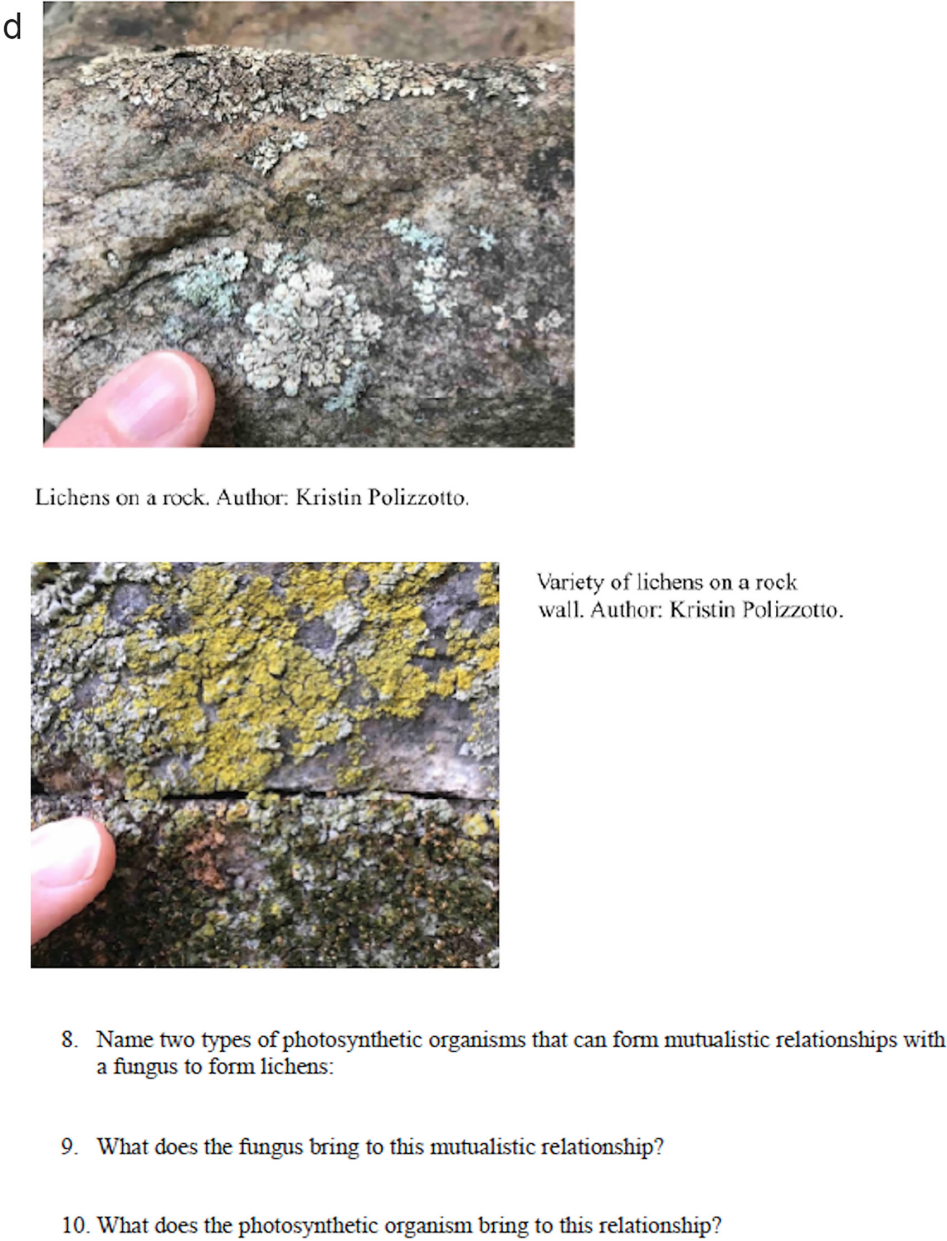
(a to d) Screenshots of four of the lab manual pages showing examples of topics covered. (a) Evolution; (b and c) Organismal biology; (d) Ecology.

It was important to us that this lab manual be provided as an open educational resource. The U.S. Government Accountability Office (GAO) report states that since 2002, college textbook prices have increased by 82% (1, 2). In the 2018 New York State Open Educational Resources (OER) City University of New York (CUNY) report (3), a Kingsborough Community College student was quoted as saying, “I can work as many jobs as I can but [the high cost of textbooks] is still going to affect me. If I'm able to pay the tuition, I won't pay for the textbooks. If I pay for the textbooks, I won't have enough money for tuition.” Access to textbooks impacts student engagement, performance, and retention at 2-year and 4-year institutions. Many students who cannot afford the lab manual and would previously use a classmate’s manual to follow the laboratory exercises no longer have that option due to the pandemic. We decided to write an OER laboratory manual that could be used in asynchronous or synchronous courses (see Fig. 1 for examples of pages). We published the manual under the Creative Commons License Agreement Attribution-Noncommercial-Share Alike 4.0 License: (https://creativecommons.org/licenses), and it is accessible via CUNY Manifold (https://cuny.manifoldapp.org/projects/general-biology-oer-laboratory-manual), the CUNY Academic Commons (https://generalbiologyoer.commons.gc.cuny.edu), and at CUNY Academic Works (https://academicworks.cuny.edu/).

## OER LAB MANUAL: CONTENT AND DEVELOPMENT PROCESS

### Content

To make the lab manual coherent, we developed each lab exercise from a phylogenetic perspective, with an emphasis on the relationship between form and function. These themes align well with general biology course learning outcomes. Most of the exercises are original and self-contained, though some include links to interactive online tools and to YouTube videos. Each lab includes the following elements:

1. Learning objectives (see Table 1)
2. An introduction to the topic
3. Keywords and definitions
4. Images, diagrams, and phylogenetic trees
5. Instructions for each activity
6. Links to short explanatory videos and websites
7. Questions, charts, etc. to complete as a lab report

### Development process

When we transitioned to online learning in March 2020, the faculty teaching the general biology laboratory set up weekly meetings to collaborate on the development of online, asynchronous lab exercises. We used the learning objectives already in place to determine what activities might help students achieve the same objectives in this new format. Each faculty member was assigned to prepare a draft of three or four exercises, which we then discussed together before posting for students. As each lab exercise was implemented, we identified issues to correct for future use.

During the 2020 summer term and fall semester, the new labs were used again in fully online courses, with continuous revisions and updates based on our experiences with the online laboratories. In addition, we created separate lab reports to facilitate submission of student work, and instructor answer keys. Finally, we added all necessary features to make the lab exercises compliant with standards for accessibility (alt-text for images, standardized headings, and subtitles for videos).

### Recommendations

Here are some suggestions that proved effective during our piloting of the OER Laboratory Manual:

1. **Laboratory guidelines.** Instructors might consider recording micro-lectures that will accompany every online laboratory activity, to give an overview of the lab and explain any potentially confusing concepts. This may help to minimize student anxiety in an asynchronous course. In a synchronous course, instructors may devote 15 minutes each week to review the instructions for each laboratory exercise.
2. **Hands-on activities and exploratory observations.** In addition to supplementary videos, instructors may ask students to safely engage in related hands-on, at-home activities, including dissections. For example, during meal preparation, students can cut fungi, fruits, vegetables, shellfish, fish, or poultry to identify structures and correlate them to their functions. Those mentioned above can relate to the following lab activities: lab activities 6 (Fungi); 8 (Plant II: Seed Plants); 9 (Animals I: Invertebrates), and 10 (Animals II: Vertebrates).

In conclusion, with the ongoing pandemic, we are pleased to share this lab manual in the hope that it will provide an inexpensive, convenient, and effective alternative to in-person labs. Additionally, our online OER laboratory manual has opened the opportunity for a second-semester general biology laboratory course to be offered online post-pandemic.
